# Detection of Her-2/neu expression in gastric cancer: Quantitative PCR versus immunohistochemistry

**DOI:** 10.3892/etm.2014.1982

**Published:** 2014-09-22

**Authors:** GUANG-JUN ZHU, CHUN-WEI XU, MEI-YU FANG, YU-PING ZHANG, YANG LI

**Affiliations:** 1 Department of Central Laboratory, The 281st Hospital of the PLA, Qinhuangdao, Hebei 066105, P.R. China; 2 Department of Pathology, The General Military Hospital of Beijing PLA, Beijing 100700, P.R. China; 3 Department of Integrated Chinese Traditional Medicine and Western Medicine, Zhejiang Cancer Hospital, Hangzhou, Zhejiang 310021, P.R. China; 4 Department of Pathology, Weifang People’s Hospital, Weifang, Shandong 261041, P.R. China; 5 Department of Oncology, The General Military Hospital of Beijing PLA, Beijing 100700, P.R. China

**Keywords:** quantitative polymerase chain reaction, gastric cancer, Her-2/neu

## Abstract

The aim of this study was to compare quantitative polymerase chain reaction (qPCR) with immunohistochemistry (IHC) for the detection of Her-2 in gastric cancer, and to investigate the correlation between the expression levels of human epidermal growth factor receptor 2 (Her-2) and clinical features. Clinical data from 426 cases of gastric cancer were collected. Her-2 expression levels in cancerous tissue were detected using IHC, and the Her-2/neu gene expression levels were determined by qPCR. The correlation between the expression level of Her-2 and clinical features was investigated. The positive expression rate of Her-2 in cancerous tissue detected using qPCR and IHC was 11.17% (46/412) and 13.38% (57/426), respectively. The positive expression of the Her-2 protein/gene was significantly correlated with the depth of invasion and lymphatic metastasis, as well as the TNM stage (P<0.05). No significant correlation was identified between positive expression of the Her-2 protein/gene and tumor location, age, gender, differentiation degree and Lauren classification (P>0.05). The diagnostic consistency was good between the two methods (κ=0.828). The results indicate that the expression of Her-2/neu is closely associated with the development of gastric cancer. qPCR is a convenient, objective and efficient method, which may be used as an alternative to IHC or fluorescence *in situ* hybridization for the detection of Her-2/neu gene.

## Introduction

Gastric cancer is the most common malignant tumor of the digestive system and it is the second most lethal cancer worldwide (1). The prevalence of gastric cancer differs regionally, and ~70% of cases occur in developing countries (2–4). The human epidermal growth factor receptor 2 (Her-2) is a member of the epithelial growth factor receptor (EGFR) family, the amplification of which may induce the overexpression of EGFR. Once it is bound to its ligand, Her-2 is phosphorylated and its function as a tyrosine kinase is activated, thus promoting cell proliferation (5). Park *et al* (6) demonstrated that Her-2 is an independent prognostic factor of gastric cancer. Her-2 is usually detected using immunohistochemistry (IHC) and fluorescence *in situ* hybridization (FISH), which each have advantages but also disadvantages. Therefore, the investigation of novel methods is necessary. In the present study, 426 cases of gastric cancer, all pathogenically confirmed, were collected. The clinical data were retrospectively reviewed. Her-2 expression in tumor tissue was examined using IHC, and the Her-2/neu gene expression was examined by quantitative polymerase chain reaction (qPCR). The aim of this study was to provide a novel method for the detection of Her-2/neu gene in gastric cancer tissues.

## Materials and methods

### Samples

Data was collected from patients admitted to the General Military Hospital of Beijing PLA (124 cases), the 281st Hospital of the PLA (107 cases), Zhejiang Cancer Hospital (99 cases) and Weifang People’s Hospital (96 cases) between 2011 and 2013. Written informed consent was obtained from all patients prior to their participation in the study. All patients were pathologically diagnosed with gastric cancer and Her-2 protein expression was detected using IHC. None of the patients received preoperative treatment with chemotherapy, radiotherapy or immunotherapy. Samples consisted of 424 cases of adenocarcinoma (including papillary adenocarcinoma, tubular adenocarcinoma, mucous adenocarcinoma and signet ring cell carcinoma), one case of gland scale cancer and one case of squamous carcinoma. The patients included 149 cases of intestinal type, 244 cases of diffuse type and 33 cases of mixed/unknown. The ages of the patients ranged between 27 and 84 years (median age, 59.2 years) and included 322 males and 104 females. According to the World Health Organization criteria (7), there were 192 poorly differentiated, 161 moderately differentiated and 73 highly differentiated cases. A total of 310 cases were observed with lymph node metastasis and 116 cases without lymph node metastasis. There were 93 cases in the cardia, 180 cases in the antrum and 153 cases in the stomach body. The cancer was classified as stage I in 40 cases, stage II in 108 cases, stage III in 248 cases and stage IV in 30 cases, respectively, according to the TNM Cancer Staging System of the American Joint Committee of Cancer (8).

### Materials and reagents

The DNA extraction kit was purchased from Qiagen (Hilden, Germany) and a Her-2/neu FISH testing kit was obtained from Beijing ACCB Biotech Ltd.(Beijing, China). The Mx3000P qPCR system was obtained from Stratagene (La Jolla, CA, USA).

### DNA extraction

DNA extraction was performed using the QIAamp DNA FFPE Tissue kit (Qiagen). The tissue was sectioned into slices (10 μm). The concentration and purity of the DNA was then measured in accordance with the manufacturer’s instructions.

### qPCR

The PCR reaction volume (20 μl) included 0.3 μl Taq DNA polymerase, 0.4 μl substrate dNTP, 2.4 μl Mg^2+^, 2.0 μl buffer and 3.0 μl DNA. The primers were obtained from the real-time PCR kit used and their catalogue numbers were Q/HDYKB007. The cycling conditions were as follows: 95°C for 5 min, followed by 45 cycles of 95°C for 30 sec, 60°C for 30 sec and 72°C for 45 sec, for a total of 40 cycles.

### 3 analysis

Her-2 gene was amplified with dual-color FISH (Her-2 gene real-time PCR kit, Guangzhou LBP Medical Science Technology Co., Ltd., Guangzhou, China) in accordance with the manufacturer’s instructions. Briefly, hybridization buffer, a DNA probe and purified water were centrifuged and then heated to 65°C overnight in a water bath. Tissue sections (4 μm) were placed on slides and immersed in a denaturing bath (2× SSC) for 5 min at 73°C, followed by dehydration in increasing ethanol concentrations and then dried. The slides were incubated with the probe at 42°C for 30 min. The slides were then washed with 0.4× SSC/0.3% NP-40 for 2 min, air-dried in the dark, counterstained with 4′,6-diamidino-2-phenylindole (DAPI) and covered with a cover-slip. The slides were observed under an Olympus BX51 fluorescence microscope (Shanghai Pooher Photoelectric Technology Co., Ltd., Shanghai, China) equipped with a digital camera. A cell was considered to be amplified when a definite cluster of >10 signals for Her-2 was found. Known positive and negative cells were used as controls for each FISH assay. Gene amplification was scored when ≥20 cancer cell nuclei exhibited a Her-2/CEP17 ratio ≥2, or when a Her-2 signal cluster was observed (9).

### IHC scoring

The 4 μm thick tissue sections of malignant tumor cells on the slides were stained with brown staining (Zymed Corporation, Inc., San Francisco, CA, USA). Briefly, sections were deparaffinized in xylene and rehydrated in grade alcohols. The antigen retrieval was performed using the wet autoclaving method in the presence of citrate buffer pH 6.0. The sections were incubated overnight in primary antibody at a dilution of 1:100 in blocking buffer at 4°C. The sections were stained using a Polin-2 plus Polymer HRP Detection System(ZSBIO, Beijing, China). Strong brown staining in the cell membrane of malignant tumor cells indicates positivity in this staining method. The HercepTest™ Interpretation Guide (10) was used to grade the membrane staining. The staining was scored as negative (0) when no membrane staining was observed or when membranes were stained in ≤10% of tumor cells, weakly positive (+) if the focal membrane was stained in ≥10% of tumor cells, intermediately positive (++) if complete membranes were weakly-moderately stained in ≥10% of tumor cells and strongly positive (+++) if complete membranes were intensely stained in ≥10% of tumor cells.

### Statistical analysis

Data were calculated using SPSS software, version 19.0 (SPSS, Inc., Chicago, IL, USA). χ^2^ and Fisher’s exact tests were used to test for an association between Her-2 amplification or protein overexpression and clinicopathological parameters. The kappa test was used to measure the consistency. P<0.05 was considered to indicate a statistically significant difference.

## Results

### Correlation between Her-2 and clinicopathological parameters in gastric cancer measured using IHC

Using IHC analysis, the rate of overexpression of Her-2 in cancerous tissues was found to be 13.38% (57/426). The overexpression of Her-2 was significantly correlated with the depth of invasion, lymph node metastasis and TNM stage (P<0.05), and no significant correlation was identified between the overexpression of Her-2 and age, gender, tumor location, differentiation degree and Lauren classification (P>0.05; Table I).

### Correlation between Her-2/neu expression and clinicopathological parameters in gastric cancer analyzed using qPCR

**Figure 1:**
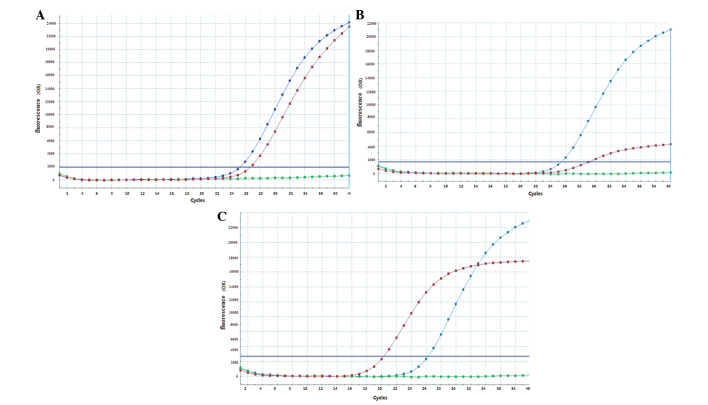
Results of quantitative polymerase chain reaction. (A) Internal control response curve. Red dots represent the internal control (Ct=26.8), blue dots represent positive control (Ct=25.3), green dots represent negative control (Ct=0). (B) Detection of Her-2, negative response curve. Red dots represent sample (Ct=29.0), blue dots represent positive control (Ct=25.5), green dots represent negative control (Ct=0). (C) Detection of Her-2, positive response curve. Red dots represent sample (Ct=29.0), blue dots represent positive control (Ct=25.5), green dots represent negative control (Ct=0). Her-2, human epidermal growth factor receptor 2.

Using PCR analysis, the positive expression rate of Her-2/neu in cancerous tissue was found to be 11.17% (46/412). The expression of Her-2/neu was significantly correlated with the depth of invasion, lymphatic metastasis and TNM stage (P<0.05), and no significant correlation was identified between the positive expression of Her-2/neu and age, gender, tumor location, differentiation degree and Lauren classification (P>0.05; Fig. 1A–C; Table II).

### Clinical pathological parameters of gastric cancer cases with relative Her-2/neu gene copy number >2 and <4.5

**Figure 2:**
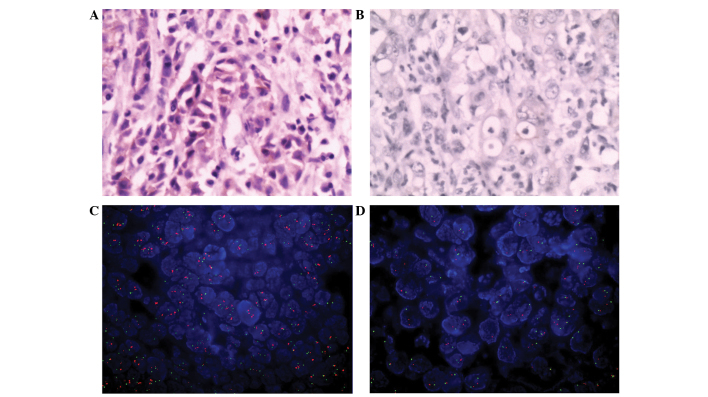
Expression of Her-2 analyzed using (A and B) immunhistochemistry and (C and D) FISH staining. (A) Positive and (B) negative expression of Her-2 protein analyzed using immunhistochemistry. (C) Amplification (case 3) and (D) nun-amplification (case 5) of Her-2 gene. Her-2, human epidermal growth factor receptor 2; FISH, fluorescence *in situ* hybridization.

There were 14 cases with a relative Her-2/neu gene copy number >2 and <4.5, the definite judgment for which was challenging. This range is used to determine whether the Her-2 gene is amplification-positive or negative. A Her-2 gene copy number >4.5 in the DNA sample that will be detected indicates an amplification-positive Her-2 gene and a value <2 an amplification-negative gene. Alternatively *in situ* hybridization can be used to detect the Her-2 gene without the use of the copy number. These comprised 9 females and 5 males. All cases were TNM stage II–III, including 3 cases of IIa, 5 cases of IIb, 4 cases of IIIa and 2 cases of IIIb. The expression of Her-2 was determined in 13 cases using IHC, and expression of Her-2/neu was determined in 8 cases using FISH (Table III; Fig 2A and B).

### Correlation between Her-2/neu gene expression and the overexpression of Her-2 protein

Her-2 overexpression was observed in 53 cases (12.86%) using IHC examination, Her-2/neu positive expression was observed in 46 cases via qPCR. In the kappa test, good consistency is indicated when 0.4<κ≤1 and poor consistency is indicated when 0≤κ≤0.4. For the studied population, there was a good consistency of diagnosis between IHC and qPCR (κ=0.828; P<0.001; Table IV).

## Discussion

Her-2/neu is located on human chromosome 17q21 and is a member of the epidermal growth factor receptor (EGFR) family. Her-2/neu is a tyrosine kinase transmembrane glycoprotein with a molecular weight of 185 kDa. It is involved in a variety of biological activities of tumor cells, including cell proliferation, adhesion, metastasis and differentiation (11).

The Her-2/neu gene has been demonstrated to be an important prognostic indicator in patients with breast cancer, as a prognostic factor for chemotherapy response and the target for trastuzumab therapy (12). At the annual meeting of the American Society of Clinical Oncology in 2009, Bang *et al* (13) reported the results from the To-GA multi-center randomized controlled clinical trial. This was the first clinical trial of targeted therapy proven to prolong survival time of patients with advanced gastric cancer, which opened a new chapter of targeted therapy for advanced gastric cancer. It laid the foundation of Her-2/neu gene detection in the diagnosis and treatment of gastric cancer and supports the use of trastuzumab in gastric cancer therapy. Based on the results from that trial, Herceptin (trastuzumab, was approved for the treatment of advanced gastric cancer in January 2010.

Previous studies have shown that >30% of human tumors overexpress the Her-2/neu gene, including breast cancer, ovarian cancer, endometrial cancer, fallopian tube cancer, stomach cancer and prostate cancer. In breast cancer, its overexpression is ~20–40% (14), whilst gene or protein expression of Her-2/neu in gastric cancer varies from 6 to 35% (15–18). Her-2/neu expression is affected by numerous factors, including tumor location, histology and specimen types.

The present study indicated that the rate of Her-2 protein expression analyzed using IHC was 13.38% (57/426) in gastric cancer. In addition, Her-2 protein overexpression was found to be significantly associated with the depth of tumor invasion (P=0.017), lymph node metastasis (P=0.016) and TNM staging (P<0.001), but not with age, gender, tumor location, the degree of differentiation or Lauren classification (P>0.05). The positive expression rate of Her-2/neu gene was 11.17% (46/412). Similarly, its expression was significantly associated with the depth of tumor invasion (P=0.012), lymph node metastasis (P=0.038) and TNM staging (P<0.001), but was not found to be associated with age, gender, tumor location, the degree of differentiation or Lauren classification (P>0.05).

IHC is currently the most commonly used method for Her-2 detection; however, FISH is also used. Although IHC is simple and cheap, its results are influenced by tissue fixation, the quality and origin of Her-2 antibody and the bias of the observer, which results in poor sensitivity and specificity. FISH is accurate, with a higher sensitivity and specificity; however, it is complex, time-consuming and has a high failure rate. In the present study, qPCR was used to detect Her-2 in tumor samples and the results were compared with the IHC results. It was found that the results from the qPCR were comparable with those from IHC (κ=0.828).

Barberis *et al* (19) demonstrated that these two methods have similar results for the detection of Her-2 in breast cancer, and are cost-effective compared with other PCR methods approved by the Food and Drug Administration. Bossard *et al* (20) obtained similar findings, indicating that qPCR is an alternative method for Her-2/neu gene detection. Tse *et al* (21) demonstrated that PCR sensitivity and specificity for Her-2 was 87.5 and 100%, respectively, with reference to IHC, while the sensitivity and specificity was 89.5 and 92%, respectively, with reference to FISH. Nistor *et al* (22) reported that the concordance rate of PCR and FISH was 92%. It has been demonstrated that Her-2/neu can be amplified and accurately detected in the paraffin tissues from breast cancer patients (23,24). qPCR is able to detect 46 samples at a time by Mx3000P qPCR, which meets the requirements of clinical laboratories. Inconsistencies between the results of these two methods are likely to be due to the process of sample collection and preparation. In terms of the factors discussed above, however, PCR is better.

In conclusion, qPCR is simple, objective, efficient and highly reproducible. The results of qPCR are consistent with the results obtained using IHC and FISH, with significant cost advantages. Therefore, it may be an alternative method for Her-2/neu gene detection in gastric cancer in the future.

## Figures and Tables

**Table I:** Expression of Her-2 measured using immunohistochemistry in cancerous tissue and clinicopathological parameters.

		Expression level of Her-2			
				
Clinical features	Number of cases	++/+++	−/+	χ^2^	P-value
Age				2.054	0.152
≤60 years	202	22	180		
>60 years	224	35	189		
Gender				0.933	0.334
Male	322	46	276		
Female	104	11	93		
Tumor location				1.523	0.467
Cardia	93	9	84		
Antrum	180	27	153		
Gastric body	153	21	132		
Differentiation degree				4.304	0.116
Poor	192	25	167		
Moderate	161	27	134		
High	73	5	68		
Lauren classification				5.047	0.080
Intestinal type	149	27	122		
Diffuse type	244	25	219		
Mixed type/unknown	33	5	28		
Depth of invasion				5.732	0.017
Serosal invasion-negative	55	13	42		
Serosal invasion-positive	371	44	327		
Lymphatic metastasis				5.782	0.016
Yes	310	49	261		
No	116	8	108		
TNM stage				44.761	<0.001
I	40	0	40		
II	108	4	104		
III	248	39	209		
IV	30	14	16		

Her-2, human epidermal growth factor receptor 2.

**Table II:** Expression of Her-2/neu measured using quantitative polymerase chain reaction in cancerous tissue and clinicopathological parameters.

		Expression of Her-2/neu			
				
Clinical features	Number of cases	Positive expression	Negative expression	χ^2^	P-value
Age				1.480	0.224
≤60 years	196	18	178		
>60 years	216	28	188		
Gender				1.794	0.180
Male	317	39	278		
Female	95	7	88		
Tumor site				1.246	0.536
Cardia	89	7	82		
Antrum	174	21	153		
Gastric body	149	18	131		
Differentiation degree				3.834	0.147
Poor	184	18	166		
Moderate	155	23	132		
High	73	5	68		
Lauren classification				3.433	0.180
Intestinal type	142	21	121		
Diffuse type	240	21	219		
Mixed type/unknown	30	4	26		
Depth of invasion				6.352	0.012
Serosal invasion-negative	51	11	40		
Serosal invasion-positive	361	35	326		
Lymphatic metastasis				4.285	0.038
Yes	296	39	257		
No	116	7	109		
TNM stage				50.034	<0.001
I	40	0	40		
II	100	3	97		
III	242	29	213		
IV	30	14	16		

Her-2, human epidermal growth factor receptor 2.

**Table III:** Clinicopathological parameters of gastric cancer cases with relative Her-2/neu gene copy number >2 and <4.5.

Case index	Gender	Lauren classification	TNM stage	IHC	FISH
1	Female	Intestinal type	IIIb	−	−
2	Female	Intestinal type	IIIa	++	−
3	Male	Diffuse type	IIIb	+++	+
4	Female	Intestinal type	IIIa	++	−
5	Male	Diffuse type	IIb	+	−
6	Male	Intestinal type	IIa	++	−
7	Female	Mixed type	IIa	++	+
8	Female	Intestinal type	IIb	+++	+
9	Female	Diffuse type	IIb	++	+
10	Male	Mixed type	IIb	++	−
11	Female	Mixed type	IIb	++	+
12	Female	Intestinal type	IIIa	++	+
13	Male	Diffuse type	IIa	++	+
14	Female	Intestinal type	IIIa	++	+

Her-2, human epidermal growth factor receptor 2; IHC, immunohistochemistry; FISH, fluorescence *in situ* hybridization.

**Table IV:** Comparison between immunohistochemistry and qPCR for diagnosis of gastic cancer.

		qPCR			
				
Immunohistochemistry	Number of cases	Positive expression	Negative expression	P-value	κ-value
++/+++	53	42	11	<0.001	0.828
−/+	359	4	355		

qPCR, quantitative polymerase chain reaction.

## Abbreviations

Her-2: human epidermal growth fact or receptor 2
EGFR: epithelial growth factor receptor
PCR: polymerase chain reaction
FISH: fluorescence in situ hybridization
